# A failed review of CCE site inspection standards and processes

**DOI:** 10.1186/s12998-019-0270-y

**Published:** 2019-10-30

**Authors:** Stanley I. Innes, Charlotte Leboeuf-Yde, Bruce F. Walker

**Affiliations:** 1 College of Science, Health, Engineering and Education, Murdoch University, Murdoch, Australia; 2 Institute for Regional Health Research, University of Southern Denmark, DK-5000 Odense, Denmark

**Keywords:** Chiropractic, Accreditation, Evaluation, Educational research

## Abstract

**Background:**

Accreditation of educational programs involves an independent agency assessing quality against a set of defined standards. Site inspection teams are appointed by an accrediting agency and compile a report with the intention of identifying deficiencies and making recommendations for their rectification and continued improvement. For chiropractic programs accreditation is carried out by Councils on Chiropractic Education (CCEs). However, the reliability of their site inspection teams remains unknown. Recent research has suggested that variability in chiropractic practice may be partially traced back to the education provider. This raises the possibility of deficient accreditation procedures that may include unsatisfactory site inspection standards or processes or the accreditation standards by which they work to.

We sought to compare the various CCEs documented standards and processes for site inspection teams for similarities and differences with the intent of making recommendations to create uniform and high quality standards. Further, we sought to compare a sample of CCEs site inspection team surveys / reports for commonly identified recommendations and quality improvements and determine if they are adequately described in their accreditation standards.

**Method:**

In December of 2018 invitation emails were sent to 4 CCEs through their website portals outlining a proposed study investigating site inspection teams’ standards and processes. Access was requested to all appropriately redacted documentation relating to site inspection teams and their chiropractic program reports. Follow up emails were sent several weeks later.

**Results:**

Only one of four of the CCEs responded by providing the requested information.

**Conclusion and recommendations:**

Three CCEs did not cooperate with this educational research. The possible reasons for the non-engagement is discussed.

**Electronic supplementary material:**

The online version of this article (10.1186/s12998-019-0270-y) contains supplementary material, which is available to authorized users.

## Background

The number of chiropractic education programs has increased considerably over the past decades. Some are run as private initiatives, some are included in university structures, sometimes even within a medical school setting. Concomitantly, perhaps both as a result and a driver, evidence-based practice approaches have become important and the traditional chiropractic *vitalistic* approaches downgraded, at least ‘officially’.

Accreditation standards and inspection procedures have been established worldwide by Councils on Chiropractic Education (CCEs) to safeguard standards and to ensure harmonisation between schools and geographical regions. CCEs are variously mandated and exist for the purposes of assuring educational quality and institutional integrity to governments, regulatory bodies, chiropractic programs, professional organizations, students and the public at large. Members of the CCEs may be elected or appointed. Medical education has adopted science and evidence-based practice as a basis for training [1]. This is not always the case for chiropractic education. Some CCE accredited chiropractic programs have aligned with an evidence-based mainstream healthcare approach and declared that since there is no evidence for vitalist or subluxation beliefs it has no place in chiropractic training, except from as a historical context [2]. Other CCE accredited colleges have continued to hold to a *vitalist* philosophy. Some even openly advertise vitalistic statements such as the New Zealand College of Chiropractic *“To offer well-resourced, integrated, relevant, evidence based curriculum with the correction of vertebral subluxation as the primary chiropractic aim”* [3] and *“The purpose of chiropractic is to help people reach and maintain excellent health and wellbeing through the care of the spine and nerve system”* [4]*.*

This has implications for patient safety and quality of care. Vitalist or subluxation trained chiropractors have been characterised by excessive X-ray usage, anti-vaccination beliefs, and poor levels of inter-professional / disciplinary communication [5, 6].

This raises the two questions,

i. How can these vitalistic educational practices occur?
ii. Why are these issues not discovered and dealt with during the CCE inspections?

In our opinion, this suggests two possibilities; chiropractic accreditation standards are not addressing such issues adequately or there is inadequate monitoring and site inspection processes. Site inspection teams, appointed by the accrediting agency, is the mechanism by which program performance is assessed against prescribed standards. Such teams compile a report with the intention of identifying deficiencies and making recommendations for their rectification and continued improvement.

We have previously conducted a systematic review of these accreditation standards, and made a series of recommendations with the intention of improving their uniformity and quality [7]. The intent of this study was to conduct a similar systematic review of the site inspection standards and processes to the same end.

Medical education is frequently ‘overseen’ by accreditation agencies, whether governmental or private. This activity rests on (among other things) the relevance of inspection, the skills and knowledge of the inspection teams, and the subsequent use made of any recommendations for improvement [8]. Obviously, accreditation and re-accreditation surveys depend on the expertise of the people involved and are therefore at risk of becoming subjective and even invalid. Historically the reliability of inspection team surveys has been unknown and difficult to study [9]. Nonetheless it is recognised as an important area for further attention as it is under-investigated [10–12].

Research to date has identified several factors that are thought to increase the likelihood of improved outcomes of this process. These include having the processes for inspection surveys clearly outlined, standardized and consistently applied to the accreditation standards [9], strong communication skills within an experienced team and team members should be temporary or replaceable so that it promotes allegiance to the accrediting organisation [11]. Also the teams should undergo detailed training and mentoring [13]. Yet another paper suggested that team members should have extensive experience in the profession, with a minimum of experience in high managerial positions (ranging from 2 to 5 years), and profession-specific certification [8].

Intrinsic to the accreditation process of chiropractic, as performed by the various CCEs, is the site inspection team appointment, training, co-ordination, quality control and review, and implementation of the survey team’s final report. However, we could not find any previous work on the monitoring aspect of the tasks of the CCEs, nor with respect to site visitations for accreditation or re-accreditation purposes of chiropractic programs in PubMed, Scopus or Chirolndex databases.

Clearly, regardless of how good the accreditation standards are, unless the monitoring process is relevant, consistent and effective, they will not be reinforced in teaching institutions that, prefer to deviate in other directions.

In view of these problems, we wanted to see if there is an appropriate and comprehensive approach to inspecting chiropractic programs for re-accreditation by site inspection / surveys by CCEs.

### Aim

The aim of this systematic audit was to investigate similarities and differences between the various CCEs inspection site team documentation and processes and compare these to known quality standards and the available evidence.

### Objectives

The objectives were to:

1. Review and compare the available site team inspection documentation from each CCE to look for similarities and differences.
2. Review and compare a sample of CCEs site inspection team surveys / reports for commonly identified recommendations and quality improvements and determine if they are described in their respective accreditation standards.
3. Make recommendations that would create a high-quality set of site inspection team standards and processes that is consistent with known best standards and evidence.

## Methods and analysis

Ethics approval was obtained from Murdoch University Human Research and Ethics Committee (2018/238) for this study.

We intended to conduct a systematic audit to investigate the three objectives.

### Eligibility criteria

The World Health Organization recommends the CCE-International as the source of information regarding evaluation of chiropractic education [14]. Consequently, we included all those CCEs who were members in good standing. The CCE-USA was a member in good standing of the CCE-I since inception until 2015 and is home to the largest number of chiropractic programs [7]. Also, the CCE-USA Accreditation Standards were released in 2013 and remain current [15]. Consequently, the CCE-USA was included in the analysis.

Thus four CCEs were included in total; Council on Chiropractic Education Australasia (CCE-Australasia) [16], Council on Chiropractic Education Canada (CCE-Canada) [17], the Council on Chiropractic Education (CCE-USA) [15], and the European Council on Chiropractic Education (ECCE) [18]. A fifth CCE was identified, the Council on Chiropractic (International) CCE-I which is a federation of the other four CCEs. The CCE-I does not conduct site inspections and was not included.

### Invitations

An email invitation with the study information was sent to the four CCEs (CCE-A, CCE-C, CCE-USA, ECCE) via their website portal in mid-December 2018 (Additional file 1). A follow up email was sent to the non-responders in mid-January of 2019.

### Data extraction process and synthesis of results

#### Objective 1. A systematic audit of CCE site inspection team documentation and processes

Method: All CCEs were approached and asked for copies of their documentation related to site inspection of chiropractic programs. CCEs were asked to de-identify all data using redaction. Once obtained these data would be further scrutinized to ensure no identifying information was found. The data would then be recorded and tabulated for a comparative analysis. This method had been used in three previous systematic reviews investigating CCEs accreditation standards [19–21]. For these studies the documentation used in the analysis was obtained because it was accessible to the public from CCE websites. The table format to compare for similarities and differences was to be structured to identify similarities and differences with respect to the following elements;

i. Team composition
ii. Team selection criteria for chiropractors and consumer members
iii. Team training / instruction
iv. Report construction

The findings were to be compared to inspection team survey documentation from another widely known and recognised Medical Accreditation organisation, such as the Australian Medical Council [22] or the Accreditation Council for Graduate Medical Education [23].

This was to allow for the identification of similarities and differences in CCEs site inspection standards and processes.

#### Objective 2. A thematic analysis of CCEs site inspection team final reports

Method: We sought to obtain copies of CCEs Site Inspection team final reports of chiropractic programs for the last 5 years from CCE-Australasia, CCE-Canada, CCE-USA and the ECCE. The reports were to be coded to identify themes and de-identified to ensure confidentiality.

Two researchers would independently review the reports and place each recommendation made by the site inspection team under the appropriate Accreditation Standard.

The two researchers would then compare their decisions for report recommendation placement and then discuss any differences. A third reviewer was available to resolve any instances when a recommendation could not be placed.

The process allows an understanding of whether the inspection team reports are comprehensive and consistent when compared to the accreditation standards. Identified ‘gaps’ could inform future iterations of accreditation standards as well as CCEs training of site inspection teams.

#### Objective 3. Recommendations for creation of a high-quality set of site inspection team standards and processes

By conducting this comparative process, and considering the evidence, similarities and differences between CCEs and other quality non-chiropractic inspection standards and processes could be identified enabling the proposing of recommendations to create uniform and high quality international set of site inspection standards, report construction and processes.

## Results

Responses were received from 3 of the 4 CCE organisations.

The CCE-USA referred us to their website and declined to forward any further material. Information on their website related to site inspection standards and processes but did not include training or recruitment data. This CCE also declined to provide site inspection team reports, as they deemed these reports to be disclosed at the discretion of the chiropractic program (CP) only. It was also claimed that this was to protect the confidentiality of CCE members, CCE office staff and CPs, and to comply with the overarching bodies’ policy of not making accreditation activities open to the public for release.

The CCE-Canada did not respond to our request despite further correspondence.

The CCE-Australasia informed us that this material was confidential in nature and could not be reproduced or commented on in any public domain or format. Also, this CCE took the view that site inspection reports were confidential and released only at the discretion of the CP. Further that this related to the integrity of the accreditation process as it depends, in part, on the Council and the Committee maintaining confidentiality with all aspects of the process other than the reporting of procedure and decisions. We recontacted this CCE and asked them to reconsider their decision on the grounds that their overarching government organisation had a policy on research and this area of investigation was identified as a high priority. Also that this study would meet all the confidentiality and privacy requirements and was redacted and not being released to the public but to researchers. The CCE-Australasia responded “*The CCE has considered your response and we re-confirm our original position as advised previously*”.

The ECCE readily complied and forwarded all site inspection documentation, including their training information. This also included site inspection team reports that were, in fact, also available on their website.

This resulted in a complete and usable set of desired documents from only 1 of the 4 CCEs.

## Discussion

### Summary of findings

We contacted four CCEs with a letter explaining a study to investigate CCEs site inspection team standards, processes, and documentation as well as seeking copies of reports from chiropractic program inspections. This letter presented the reasons and rationale for the study, appropriate ethics approval, and addressed the sensitive area of confidentiality and anonymity. Only 1 of 4 CCEs responded positively and agreed to participate.

### Potential explanations

The unwillingness to participate is puzzling as the 4 CCEs are undertaking the same task, with the same goals, using supposedly uniform standards. So, while one of the CCEs was completely transparent the other three were not at all. Clearly, this makes it difficult for the CCEs to compare and align activities between themselves. This compares poorly with a similar study of Dental programs in Australia in 2007, where such inspection team reports were readily made available using the same research methodology [24].

We considered several possible explanations for the non-participation of the CCEs. First, is that these organizations do not differentiate between the public and researchers. When we made this differentiation on a repeat request one of the non-participating CCE did not change its mind. Obviously the CCE had control over ensuring that confidentiality requirements were met as they were invited to remove any sensitive information before forwarding the required documentation. Perhaps there was a distrust of the authors by the CCE or an overcautiousness that resulted in a decision to be wary despite the authors having University Ethics approval.

It is tempting to speculate that these responses of ‘silence’ and ‘public confidentiality’ were stimulated by a lack of confidence in their own standards and processes for site inspection of the various chiropractic programs. This may have resulted in an approach to camouflage this possibility by refusing to participate. Alternatively, these organizations may be confident in their established standards and methods and have no desire to change them.

It is argued by some that transparency of accreditation findings motivates educational or healthcare programs to hide any short-comings and avoid ‘brand damage’ that would result in a reduced capacity to compete in a competitive market place [25]. This could be less important in regions where there is a greater number of student enrolments than available places in CPs.

Proponents of confidentiality hold to the view that an environment without negative public consequences improves the likelihood of disclosure by CPs to CCEs [26, 27]. The resultant openness between agency and educator produced by this ‘safe environment’ is thought to be conducive to a collaborative working relationship that increases the chances of quality improvements [28]. However, the evidence for this is elusive.

The question for us as bona fide educational researchers, is why did one of the four CCEs trust the authors and agree to fully participate while the other 3 did not? We suspect that cultural differences may be one factor as the positively responding CCE already transparently publishes inspection team reports on their website. It is also possible that there are differences between the composition of the CCEs executives that influence the willingness to subject themselves to scrutiny with the possibility of having to change and improve. Those who argue in favour of such transparency believe the public and students have a right in a democratic society to know of educational practice standards [29]. They also argue that the possibility of positive reports motivates educational programs to strive to improve themselves and increase their marketability [26, 28].

## Conclusion

We could not compare the CCE site inspection standards and processes because only one provided information. We conclude that there is a wide discrepancy in transparency between CCEs on the topic of site inspections.

Future research to develop a clearer understanding of the internal machinations and thinking of CCEs with respect to such matters may be best obtained through interviews with key personnel.

## Additional file

Additional File 1: Study Invitation (DOCX 186 kb)

## Data Availability

Not applicable

## Abbreviations

CCE: Council on Chiropractic Education
CCE-International: Council on Chiropractic Education – International
CCEs: Councils on Chiropractic Education
CP: Chiropractic Program
ECCE: European Council on Chiropractic Education
