# Supplementary figures and images for: A failed review of CCE site inspection standards and processes
Source: Chiropr Man Therap. 2019 Oct 30;27:49. doi: 10.1186/s12998-019-0270-y (PMC6820976; doi:10.1186/s12998-019-0270-y)

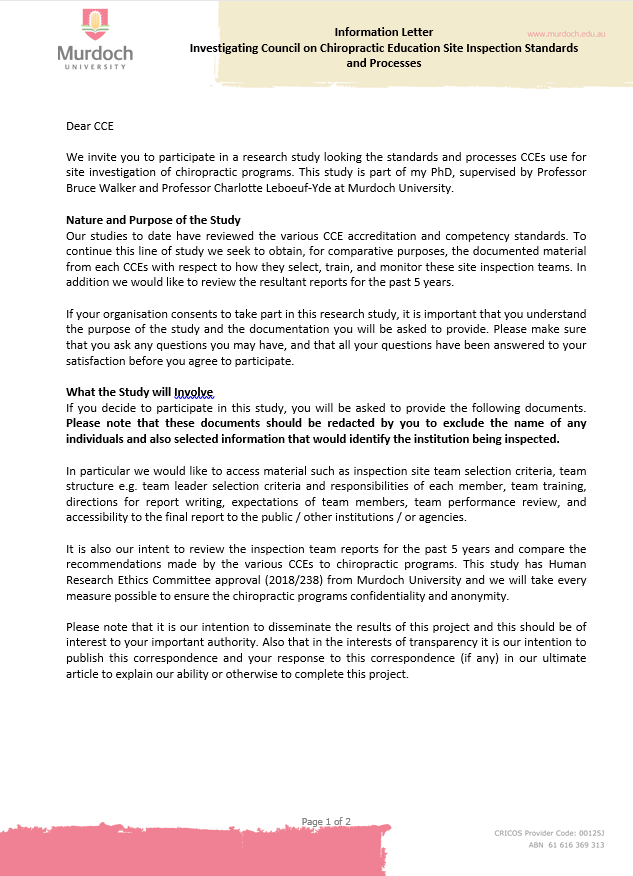

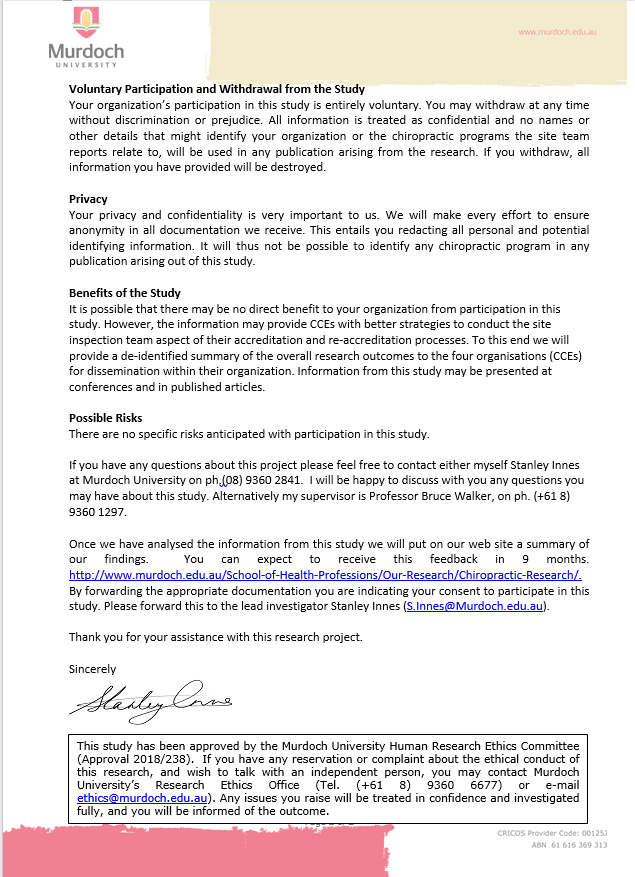

Supplement: Supplementary file 1 — Study Invitation (DOCX 186 kb) [file 12998_2019_270_MOESM1_ESM.docx]
